# Patient and carer perceptions and acceptability of current management practices in paediatric X-linked hypophosphatemia treated with burosumab therapy

**DOI:** 10.1093/jbmrpl/ziaf033

**Published:** 2025-12-06

**Authors:** Jessica L Sandy, Christine P Rodda, Aris Siafarikas, Andrew Biggin, Christie-Lee Wall, Lucy Collins, Aaron Schindeler, Peter J Simm, Craig F Munns

**Affiliations:** Institute of Endocrinology and Diabetes, The Children’s Hospital at Westmead, Westmead, NSW 2145, Australia; Faculty of Medicine and Health, University of Sydney, Camperdown, NSW 2006, Australia; Centre for Hormone Research, Murdoch Children’s Research Institute, Melbourne, VIC 3002, Australia; Department of Paediatrics, University of Melbourne, Melbourne, VIC 3010, Australia; Department of Endocrinology and Diabetes, Perth Children’s Hospital, Nedlands, WA 6009, Australia; Institute for Health Research, Notre Dame University, Fremantle, WA 6160, Australia; Medical School, Paediatrics, University of Western Australia, Perth, WA 6009, Australia; Institute of Endocrinology and Diabetes, The Children’s Hospital at Westmead, Westmead, NSW 2145, Australia; Faculty of Medicine and Health, University of Sydney, Camperdown, NSW 2006, Australia; Institute of Endocrinology and Diabetes, The Children’s Hospital at Westmead, Westmead, NSW 2145, Australia; Department of Endocrinology and Diabetes, Royal Children’s Hospital, Melbourne, VIC 3052, Australia; Bioengineering and Molecular Medicine Laboratory, The Children’s Hospital at Westmead and the Westmead Institute for Medical Research, Westmead, NSW 2145, Australia; School of Chemical and Biomolecular Engineering, The University of Sydney, Camperdown, NSW 2006, Australia; Centre for Hormone Research, Murdoch Children’s Research Institute, Melbourne, VIC 3002, Australia; Department of Paediatrics, University of Melbourne, Melbourne, VIC 3010, Australia; Department of Endocrinology and Diabetes, Royal Children’s Hospital, Melbourne, VIC 3052, Australia; Child Health Research Centre, Faculty of Medicine, The University of Queensland, Brisbane, QLD 4072, Australia; Department of Endocrinology and Diabetes, Queensland Children’s Hospital, Brisbane, QLD 4101, Australia

**Keywords:** X-linked hypophosphatemia, XLH, hypophosphatemia, burosumab, paediatrics

## Abstract

X-linked hypophosphatemia (XLH) is an X-linked dominant condition where fibroblast growth factor-23 (FGF23) excess leads to hypophosphatemic rickets, lower limb bowing, and musculoskeletal pain. Burosumab, a monoclonal antibody against FGF23, has been shown to ameliorate the clinical phenotype of XLH and has recently been approved for use in many countries. This study aimed to evaluate patient and parental/caregiver perception of burosumab therapy and the acceptability of current management practices in Australia. Children with XLH and parents/carers were invited to respond to a survey on clinical and management information including use of telehealth, access to multidisciplinary team members, and perceptions and experience regarding burosumab therapy. This was a multi-centre, cross-sectional survey-based study involving 4 tertiary Australian children’s hospitals. A total of 21 survey responses from parents/carers were received between December 2022 and October 2023. Mean (SD) age at time of survey was 12.7 (4.1) yr and median time on burosumab was 42 mo (range 2-100). Reported side effects of burosumab were limited to local skin reactions (38%, *n* = 8) and injection site pain (5%, *n* = 1), with the majority (62%, *n* = 13) reporting no side effects. Logistical issues (availability from the pharmacy or medical centre holiday closure) led to most instances of missed or delayed doses, which were reported by 24% (*n* = 5). Most participants reported seeing their specialist both face-to-face and via telehealth (64%, *n* = 14). The majority saw an endocrinologist (100%, *n* = 21) and orthopaedic surgeon (67%, *n* = 14), but only a small minority saw a psychologist (10%, *n* = 2). Answers to Likert scale questions revealed that most parents/carers and children reported a perceived improvement in physical and psychological symptoms and function with burosumab therapy. This study supports the use of recently published local guidelines to manage children with XLH on burosumab due to high satisfaction expressed by children and parents/carers. However, logistical issues leading to delayed or missed doses should be addressed.

## Graphical Abstract

**Figure:**
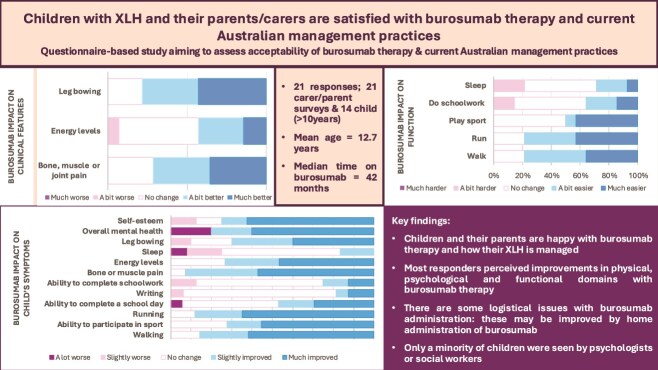

## Introduction

X-linked hypophosphatemia (XLH) is an inherited skeletal dysplasia caused by X-linked dominantly inherited mutations in the phosphate regulating endopeptidase homolog X-linked (*PHEX)* gene.^1^  *PHEX* mutations lead to an increase in the expression of circulating fibroblast growth factor-23 (FGF23), which leads to dysregulated phosphate metabolism. Individuals with XLH exhibit decreased phosphate reabsorption in the kidneys and produce excess phosphate in the urine.^1^^,^^2^ Individuals with XLH experience significant physical symptoms, such as rickets, leg bowing, short stature, dental problems, and musculoskeletal pain.^2^^,^^3^ In addition, XLH can have a negative impact on quality of life and can impart a high psychosocial burden of disease on affected individuals, including difficulties with mental health, self-esteem, bullying and feelings of isolation.^4–6^

Conventional therapy for XLH involves phosphate supplementation to overcome the phosphate depletion associated with excess FGF23 as well as administration of either calcitriol (1,25(OH)2D3) or alfacalcidol to normalize Vitamin D levels.^7^ While this intervention can curb the impact of XLH, such as improving bone abnormalities and pain and reducing the severity of dental disease,^8^^,^^9^ its overall efficacy can be limited and variable. Oral phosphate has unpleasant side effects and requires multiple daily dosing, factors that may limit adherence to therapy. In addition, conventional therapy does not normalize the hypophosphataemia, does not fully correct the clinical features of XLH, and imparts a risk of complications such as nephrocalcinosis or hyperparathyroidism.^7^^,^^10^

Burosumab is an emergent monoclonal antibody therapy for XLH that targets FGF23. A range of clinical trials in children and adults have demonstrated safety and efficacy for burosumab in managing XLH.^11–14^ One of the most notable paediatric clinical studies was a phase III randomized trial that confirmed greater clinical improvements in growth, rickets severity and biochemistry with burosumab compared to conventional therapy.^14^

In Australia, burosumab (brand name Crysvita) is now available via the public health system for the management of XLH. To be eligible, a patient must have documented confirmation of a *PHEX* pathogenic variant or have a confirmed diagnosis of XLH presenting with (1) low serum phosphate, (2) current or historical radiographic evidence of rickets, (3) elevated serum or plasma FGF23, and (4) renal phosphate wasting. Their treatment must be managed by an endocrinology or nephrology specialist. Burosumab was accepted onto the Australian Register of Therapeutic Goods in September 2021 and is increasingly being prescribed to manage XLH. Following this, Australian clinical practice guidelines were published in 2022 highlighting a favoured role for burosumab in the management of paediatric XLH.^15^ These guidelines cover topics including the recommended work-up prior to commencing burosumab, administration and dose, monitoring and adjustment of burosumab dose, the side-effect profile; management by a multidisciplinary team, and transition to adult care. Following the introduction of this novel medication and updated management protocols, it is important to assess post-release efficacy and patient satisfaction.

Consequently, this study—which spans multiple children’s hospitals in Australia—aimed to evaluate patient and parental/caregiver perception of burosumab therapy and the acceptability of current management practices in Australia. This follows a recent 2023 report characterizing the prevalence and features of paediatric XLH in Australia and New Zealand.^3^

## Materials and methods

The study was approved as a low and negligible risk protocol by Sydney Children’s Hospital Human Research Ethics Committee (2021/ETH11466). It was designed as a multi-centre prospective, cross-sectional survey-based study, taking place in the following centres: Sydney Children’s Hospital Network, Royal Children’s Hospital Melbourne, and Perth Children’s Hospital, Queensland Children’s Hospital.

Survey was designed by investigators, predominantly paediatric endocrinologists managing patients with XLH, with feedback sought from XLH Australia representatives. Survey was tested by investigators to ensure there were no errors and that the survey was readable and user friendly. Survey contained single response, multiple response and free text questions on demographics, inheritance, burosumab therapy, difficulties with injection administration, side effects, missed doses, telehealth versus face-to-face clinical encounters, and orthopaedic procedure cancellation. Likert scale questions asked about physical and psychosocial improvements following commencement of burosumab.

Participants were included if they were a carer or parent for a child with XLH who was less than 18 yr of age. In addition, affected children 10 yr or older were also invited to complete a child version of the survey. Inclusion criteria: (1) Parent/carer must have a child with confirmed XLH diagnosed by a doctor; (2) Child must be on burosumab therapy for at least 6 mo; (3) Must be able to read English and use a computer. Exclusion criteria: (1) Non-English speaking/illiterate; (2) Affected child is 18 yr or older.

Recruitment was by treating physicians who identified patients and made contact by email address obtained from the clinical file. Emails included the patient information sheets and a link to the RedCap survey. Consent was made by parents prior to proceeding; a compulsory answer to the consent from was required to access the survey. Survey contained data (postcode, date of birth and initials) to ensure there were no duplicate responses. The XLH Acceptability Survey is included in Appendix S1.

Descriptive statistical analysis was performed using IBM SPSS Statistics for Macintosh, Version 28.0 (Armon, NY). Mean ± SD was reported for normally distributed data, and median (range) for non-normally distributed data, with data distributions assessed visually and statistically (by calculating skewness and kurtosis). Likert scale answers were represented visually in 100% stacked bar charts. Free text answers were analysed categorically.

## Results

The survey was conducted over a 10-mo period (December 2022 to October 2023) with 21 parental/carer responses received out of 51 families approached (42% response rate). Fourteen related to a child >10 yr old and included child responses. The demographic information and results are summarized in Tables 1 and 2. The mean age of the child at the time of survey was 12.7 ± 4.1 (SD) yr. The median time on burosumab was 42 mo (range 2-100 mo), with all children having taken conventional therapy for at least 6 mo. About 71% (*n* = 15) were sporadic cases with no family history of XLH, 29% (*n* = 6) had a maternal history of XLH, 52% (*n* = 13) were female and 14% (*n* = 3) had an affected sibling.

**Table 1:** Parent survey results.

	**Response**
**Demographic information**	
**Mean ± SD age at time of survey**	12.7 ± 4.1 yr
**Median (range) time on Burosumab therapy**	42 (2-100) mo
**Family history of XLH, % (*n*)**	
**Sporadic cases/no family history**	71 (15)
**Maternal history**	29 (6)
**Sibling affected**	14 (3)
**Survey item**	
**Does your child prefer burosumab or oral therapy?, % (*n*)**	
**Burosumab**	90 (19)
**Oral therapy**	10 (2)
**Who gives your child’s burosumab injection?, % (*n*)**	
**Nurse at general practice**	14 (3)
**Hospital nurse**	67 (14)
**General practitioner**	19 (4)
**Would you be willing to give the Burosumab injection yourself at home?, % (*n*)**	
**Yes**	57 (12)
**Uncertain/no answer**	38 (8)
**No**	5 (1)
**Difficulties with burosumab injection, % (*n*)**	
**Yes**	24 (5)
**No**	76 (16)
**How many burosumab injections does your child receive every 2 wk, % (*n*)**	
**1**	43 (9)
**2**	52 (11)
**3**	5 (1)
**What side effects has your child experienced from burosumab?, % (*n*)**	
**Local skin reaction**	38 (8)
**Bone/joint pain**	0 (0)
**Pain at injection site**	5 (1)
**No side effects**	62 (13)
**Has your child missed any doses of burosumab?, % (*n*)**	
**Yes**	24 (5)
**No**	67 (14)
**Uncertain**	10 (2)
**Reasons for missing doses**	
***N* = 4**	Logistical issues—including availability of or ability to get medication from pharmacy or have it administered at the GP at the appropriate time.
***N* = 1**	Illness (fever)
**Does your child see your primary XLH specialist face-to-face or via telehealth?, % (*n*)**	
**Face-to-face only**	27 (6)
**Both face-to-face and telehealth**	64 (14)
**Since starting burosumab, has your child had any orthopaedic surgery cancelled or deferred as it was no longer needed?, % (*n*)**	
**No**	68 (15)
**Uncertain or no answer**	14 (3)
**Yes**	18 (4)
**Proportion of children with the following healthcare professionals involved at any point in their care, % (*n*)**	
**Endocrinologist**	100 (21)
**Nephrologist**	10 (2)
**General practitioner**	86 (18)
**General paediatrician**	24 (5)
**Orthopaedic surgeon**	67 (14)
**Neurosurgeon**	5 (1)
**Craniofacial surgeon**	0 (0)
**Rehabilitation specialist**	5 (1)
**Nurse**	81 (17)
**Physiotherapist**	62 (13)
**Occupational therapist**	5 (1)
**Psychologist**	10 (2)
**Social worker**	5 (1)

**Table 2:** Child survey results (>10 yr, *n* = 14).

**Do you prefer burosumab or injections?, % (*n*)**	
**Burosumab**	100 (14)
**Do you prefer to see your doctor face-to-face or via telehealth?, % (*n*)**	
**Face-to-face**	21 (3)
**Telehealth**	36 (5)
**Both/either/unsure**	43 (6)
**Would you or your parent be happy to give burosumab at home?, % (*n*)**	
**Yes**	57 (8)
**No**	29 (4)
**Don’t know**	14 (2)
**Side effects of burosumab, % (*n*)**	
**No side effects**	50 (7)
**Skin rash**	43 (6)
**Muscle/bone pain**	7 (1)

All but 2 parents/carers (90%, *n* = 19) indicated that their child preferred burosumab over oral therapy. Of the 2 parents who disagreed, 1 of their children still indicated a preference for burosumab. Most participants reported no side effects (62%, *n* = 13) with the most common side effects being local skin reactions (38%, *n* = 8) and a single case of injection site pain (5%, *n* = 1).

Five responders (24%) reported experiencing difficulties with burosumab. Two reported pain was an issue, and an additional 2 reported that anxiety and related behaviours were a particular difficulty for their child (1 whose issues had since resolved), 3 parents/carers stated that logistical issues were difficult, such as coordinating holidays or public holidays with injections, school leave for injections, pharmacy storage, and the need to keep burosumab refrigerated. Five parents/carers (24%) reported their child had missed a dose of burosumab; 4 of these were due to logistical issues including medical practice being closed or availability of medication at the pharmacy. One missed dose was due to illness (fever). Notably, over half of participants responded that they would be willing to give injections themselves at home (57%, *n* = 12); with much of the remainder uncertain or with no answer given (38%, *n* = 8). The same proportion of children (57%, *n* = 8) also reported that they would be willing to have their injection done at home.

The majority (67%, *n* = 14) of parents/carers reported seeing their specialists both in-person and via telehealth. Some (29%, *n* = 6) saw the specialist face-to-face only, with 1 participant not giving a response. When asked whether they preferred telehealth or face-to-face consults, the majority gave no answer or were uncertain (85%, *n* = 19); 2 (10%) preferred face-to-face and 1 (5%) preferred telehealth. All 14 children responded to this question; 4 (29%) reported no preference, 3 (21%) reported a preference for face-to-face reviews, 5 (36%) preferred telehealth, and 2 (14%) were uncertain. All participants reported seeing a paediatric endocrinologist (100%, *n* = 21), and the majority also saw an orthopaedic surgeon (67%, *n* = 14). Notably, only a small minority reported seeing a psychologist (10%, *n* = 2).

**Figure 1:**
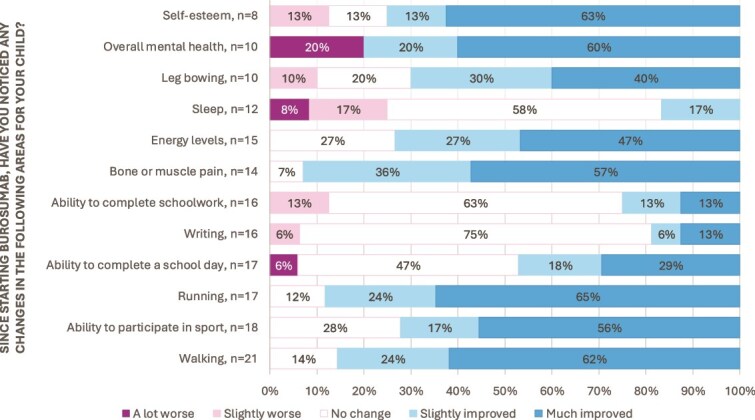
Likert scale breakdown of parental responses to changes in a range of key clinical outcomes of how a child has responded to burosumab therapy.

**Figure 2:**
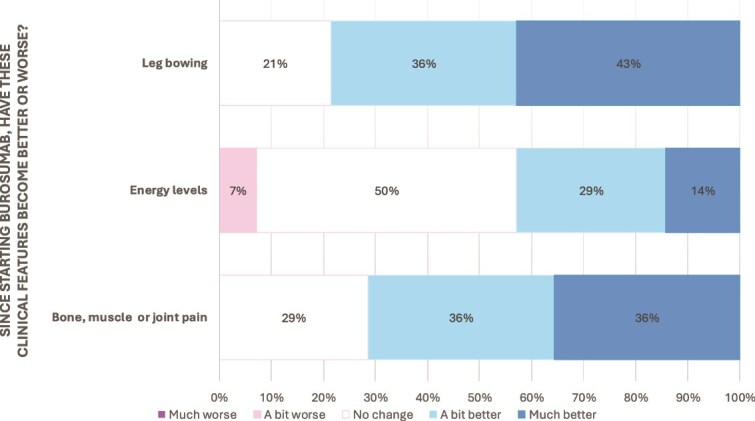
Likert scale breakdown of parental responses to changes in functional measures such as sleep, schoolwork, sport, running and walking in response to burosumab therapy.

**Figure 3:**
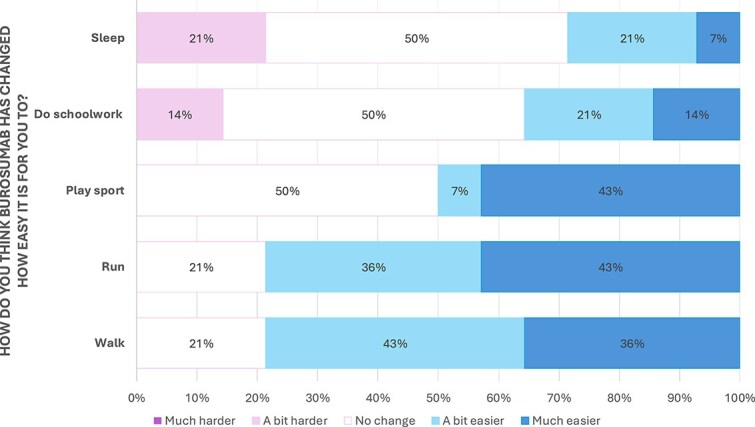
Likert scale breakdown of child self-reported responses to pain, energy levels and leg bowing in response to burosumab therapy.

Answers to the Likert scale questions on perceptions of burosumab effectiveness are shown in Figures 1-3 for both parents/carers and children. As shown, most reported a perceived improvement in physical and psychological symptoms and function because of burosumab therapy. Figure 1 shows perceived changes by parents/carers. Some responders noted technical difficulties with this question which may account for the lower response rate for this question, however, four parents/carers outlined the answers they wished to give in the free text section. Despite this, the figure demonstrates clearly that most parents/carers noted improvements across the physical and psychological symptoms. At least 70% of responses reported an improvement (“slightly improved” or “much improved”) in walking, running, sport participation, musculoskeletal pain, energy levels, leg bowing, self-esteem, and overall mental health. Over 50% reported no change in writing, ability to complete schoolwork, and sleep. Almost half of the responders reported an improved ability to complete a school day (47%, *n* = 8) with the same number (47%, *n* = 8) reporting no change. Only a small minority who reported worsening of symptoms since starting burosumab; 1 (13%) stated their child’s self-esteem was slightly worse, 2 (10%) reported mental health was a lot worse, 1 (10%) reported leg bowing was slightly worse, 2 (13%) reported ability to complete schoolwork was slightly worse, 1 (6%) reported writing was slightly worse, and 1 (6%) reported a slightly worse ability to complete the school day. One of the parents who reported a worsening in their child’s ability to complete a school day and mental health stated in the free text question that this change was likely due to factors other than burosumab (such as the pandemic, puberty, and life experiences).

Figures 2 and 3 demonstrate the responses from the children themselves regarding changes seen after starting burosumab. All children answered all questions. Overall, there were sizeable improvements seen in all areas in most children. Over 70% reported a positive change in musculoskeletal pain, leg bowing, running and walking, with none reporting any worsening of these symptoms. Energy levels, ability to play sport, ability to do schoolwork, and sleep were improved in 43% (*n* = 6), 50% (*n* = 7), 35% (*n* = 5), and 28% (*n* = 4) respectively, with another 50% (*n* = 7) noting no change.

Answers to free text question (“Do you have any other comments about burosumab therapy?”) included 7 parents/carers and 1 child with positive comments. One parent expressed a desire to be able to home administer burosumab. One parent/carer mentioned previous skin reaction (now resolved). These comments are included in Appendix S2.

## Discussion

This is a relatively small but important study of a rare disorder investigating overall acceptability of a novel therapy and current management practices. This research shows a high overall satisfaction among the Australian XLH community with burosumab therapy for children with XLH and current management practices.^15^ Consistent with clinical trial data, side effects were uncommon and mild in severity.^11^^,^^16^ The preference for burosumab over conventional therapy was extremely high and was associated with a perceived improvement in function and reported satisfaction with treatment. Notably, all participants had previously received conventional (oral) therapy and all but 1 child and 2 parent/carers indicated that burosumab therapy was preferred.

Despite the overall high level of satisfaction, this study found that logistical issues can interfere with burosumab administration. In Australia, it is often up to the family or individual to organize collection of burosumab from pharmacy and delivery to the doctor or nurse who will then administer it. This is often required for each dose, every 2 wk. As reported by some responders in this study, as well as anecdotally noted in the authors’ clinical practice, families find that their ability to travel is limited by timing of burosumab dosing, the need for burosumab to be refrigerated, and the requirement for medical professional administration. In this survey, some individuals missed or substantially delayed doses due to these logistical issues, including closure of medical practices for public holidays. These factors may be mitigated by self-administration of burosumab. Home administration of subcutaneous medications, such as insulin and growth hormone, is well accepted for other paediatric conditions, and home administration of other monoclonal antibodies has been shown to be feasible and safe.^17^ A recent study in Japan and Korea confirmed the safety efficacy of self or carer administration of burosumab in both children and adults with XLH, showing no increase in adverse events and improvement in biochemistry.^18^ However, guidelines and the consumer medication information do not currently recommend self- (or carer-) administration of burosumab.^15^ Our data suggest that this may be acceptable to families, with only 1 parent/carer indicating that they would *not* be comfortable giving injections at home compared with over half stating that they would be. Given the logistical issues outlined above, self-administration of burosumab may reduce time, money, resources, and stress associated with burosumab injections. Further research is required to assess this, to confirm the safety and efficacy of home administration for burosumab, and to assist in advocating for changes to administration requirements.

This study differs from other reports with a focus on patient approval rather than clinical outcomes or opinion. While there are many benefits of this medication evident in the literature to date,^16^^,^^19^ this study focuses on the opinion of families of burosumab therapy, showing a high rate of satisfaction and acceptability. It also presents data on subjective improvements in function, pain and energy, and mental health aspects of disease. These results contribute to the growing body of post-trial research of the benefits of burosumab therapy on psychosocial aspects and quality of life.^20–22^ However, more in depth research, including qualitative studies, would be valuable to confirm the full impact of burosumab in XLH and to better guide clinicians on how best to manage these individuals going forward.

Similar to a previous paper reporting results of the Australian and New Zealand clinician survey,^3^ there were some responders (18%, *n* = 4) who reported that orthopaedic procedures were cancelled after commencement of burosumab therapy. Burosumab has been shown to correct lower limb deformities^12^^,^^23^ but there have been no studies to date confirming that this subsequently leads to fewer orthopaedic procedures, and the implications of this from a clinical, financial and quality of life point of view. This would be an interesting and important topic for future studies to explore.

These results emphasize the importance of advocating for and encouraging patients with XLH to access psychology support. Despite known psychological impacts of XLH^5^^,^^24^ and guidelines emphasizing the importance of psychological support,^15^ only a small minority of patients reported seeing a psychologist (10%, *n* = 2). This is consistent with the literature, including a recent prevalence survey by Australian Paediatric Surveillance Unit, which found that only 3% of cases were seen by a psychologist.^3^ It is possible that, given the notable improvements in clinical manifestations as noted above, there may be less need for mental health support in patients on burosumab. However, ensuring that families can access psychosocial support if needed is essential. More research is needed to assess the reasons behind the low rates of psychology input in the management of these children, as well as to assess the impact of long term burosumab on psychosocial burden of disease.

The local Australian context is important to consider as limits generalisability to other cohorts. There is much variability across the world as to who have access to burosumab therapy. Most of the world, including most lower- and middle-income countries, does not currently have government funded burosumab for anyone, and many other countries only fund this medication for children, not adults, or those who are severely affected or not improving with conventional therapy.^25–28^ In Australia, the promise of lifelong access to government-funded burosumab is likely to impact patient experience. Similar studies in other countries would be valuable to assess the local experience of individuals with XLH to guide clinicians and policy makers.

Limitations of this study include the small size and study design. This is a small study of a rare disease; a recent prevalence survey reported 75 known cases of paediatric XLH in Australia.^3^ Therefore, while the 21 responses received were not sufficient for power calculations or in-depth statistical evaluation beyond descriptive analysis, this likely represents around 30% of the total known XLH cases, including those not on burosumab. In addition, the survey-based study design introduces a risk of selection bias, as those with stronger views on burosumab or existing protocols may be more likely to participate. It is interesting to note that 71% reported no family history of XLH. Based on the literature including the recent Australian prevalence survey,^3^ the expected proportion of sporadic mutations is around 30%. Therefore, our sample of affected children is less likely to have a parent affected by XLH, and the parent/carer responding is also less likely to be affected themselves. It is hard to predict how this may impact responses, but it is likely that those with a family history do have a different experience than those who do not. The response rate for this survey was 42%. While this is less than half, it is consistent with what is expected based on literature suggesting that an average response rate for online surveys is around 44%.^29^

In our study, the quantitative, survey-based design allowed for inclusion of a larger cohort, across more geographical locations, with minimal resources. However, future studies aiming to evaluate patient and carer perceptions may consider exploring the findings of this study in more detail with the use of different methods, for example, a qualitative, interview-based study using thematic analysis. This style of study could also clarify if whether the changes seen, both the improvements seen for most or indeed the worsening of sleep or overall mental health as seen in 1 or 2 children, were due to burosumab or other causes. This research is a cross-sectional study done at one point in time; longitudinal research would also be useful to assess continued satisfaction over time. In addition, as more children have earlier access to burosumab, patient needs and management practices may change, and these should be continued to be documented, and reflected in updating guidelines.

These data suggest several focus areas for future research. As discussed above, these include studies looking at feasibility of burosumab home-administration and assessment of ongoing satisfaction is important. Future research on the need for orthopaedic intervention and the psychosocial impacts of burosumab in XLH, such as the impact on mental health, self-esteem, and the ability to complete schoolwork or a school day, will be crucial in understanding the needs of people with XLH who have had early and continuous access to burosumab therapy. These affected individuals are likely to have a different experience of their condition compared with earlier generations, with burosumab therapy potentially leading to improvements in skeletal alignment, quality of life, physical and psychosocial function, and mental health. Understanding these improvements, and importantly what residual deficits or challenges remain, will allow us to optimise management, minimise disease impact, and improve the lives of those with XLH and their families. In addition, looking at care throughout the life of an affected individual, such as the experience of adolescents with XLH on burosumab and the healthcare transition process, would allow clinicians to gain a more holistic approach to caring for children with XLH on burosumab.

## Conclusions

These data show a high satisfaction level among children with XLH and their families with burosumab therapy and current guidelines, while also highlighting several areas for improvement, including increased use of psychological services, and reducing logistical issues associated with burosumab administration. For the latter, self- or carer-administration of burosumab is a possible solution to consider. Future research on the impact of early commencement of long term burosumab on individuals with XLH will allow for greater understanding of this condition and allow further improvements in management practices.

## Supplementary Material

Supplemental_Appendix_2_ziaf033

## Data Availability

The data underlying this article will be shared on reasonable request to the corresponding author.
