# Supplementary material for: Patient and carer perceptions and acceptability of current management practices in paediatric X-linked hypophosphatemia treated with burosumab therapy
Source: JBMR Plus. 2025 Dec 6;9(Suppl 5):v30–8. doi: 10.1093/jbmrpl/ziaf033 (PMC12723661; doi:10.1093/jbmrpl/ziaf033)
Supplement: Supplemental_Appendix_2_ziaf033 [file supplemental_appendix_2_ziaf033.docx]

**Supplemental Appendix 2**

| **Parent/carer** |
| --- |
| amazing difference between my child who has received it for 5 year and my other child who has never had it |
| It's been working well to date. We are happy with Burosumab. |
| We are very happy with Burosumab therapy! |
| It has definitely been life changing. She has no tickets, no bowed legs and the pain in her legs has subsided |
| Its much better than the doses of calcitriol and phosphorous we previously had to administer. it would great if Burosumab could be incorporated into a tablet for home administering. |
| Thank God we finally got access to it. |
| Initially [my child] had skin reactions with Burosumab, but it's slowly waning away after a year or so. |
| May be very efficient for my child . Hope she will be better, she has more energy and feel better after 2 months . After drinking phosphate and rocaltrol for 4 years my child legs are bowing so much, she feel pain a lot |
| **Child** |
| I am very happy with injections |

**Table: Responses to free text question: “Do you have any other comments about burosumab therapy?”**
